# Experimental Investigation of Additive Manufacturing of Continuous Carbon Fiber Composites with Multifunctional Electro-Tensile Properties

**DOI:** 10.3390/ma14216574

**Published:** 2021-11-01

**Authors:** Ritesh Ghimire, Frank Liou

**Affiliations:** Department of Mechanical and Aerospace Engineering, Missouri University of Science and Technology, Rolla, MO 65409, USA; liou@mst.edu

**Keywords:** additive manufacturing, 3D printing, continuous carbon fiber composites, multifunctional properties, solid laminates, tensile strength, electrical resistance

## Abstract

Manufacturing processes for monofunctional and multifunctional materials vary depending on the design optimization. Multifunctional continuous carbon fiber composites provide great potential in achieving coupled structural and electrical properties for their applications in aircraft, unmanned aircraft systems, and spacecraft. Proper optimization of tensile and electrical properties offers benefits early in the design and continuous operational safety phases to obtain coupled multifunctional properties. In this paper, fused filament fabrication additive manufacturing (AM) technique was used to fabricate continuous carbon fiber solid laminated composites test coupons. The proposed new method characterizes the electrical conductivity’s coupled effects on the tensile properties, including the failure loads and modes. This paper addresses a novel way of integrating electrical function into the composites that significantly reduce weight, potentially replacing the bulky electrical wires. Tensile and electrical conductivity tests were concurrently conducted on coupons, and the results were plotted and tabulated. The results showed the multifunctional properties of the maximum ultimate tensile strength of 392 MPa with the maximum tensile load of 8907 N, and resistance of 37.5 G·Ω. The average values for ultimate tensile strength and maximum load were 371 MPa and 8459 N, respectively.

## 1. Introduction

Additive manufacturing (AM) offers several advantages over traditional manufacturing techniques. Different AM techniques are available and the most commonly used AM technique is the fused filament fabrication (FFF) method. While the tensile strength of the 3D printed coupons was addressed, the experimental investigation of additive manufacturing of continuous carbon fiber composites as well as the electro-tensile multifunctional properties was not included [1]. An open-source FFF printer was used for 3D printing short fiber reinforced nylon filament to study the effects of low-temperature thermal annealing on the 3D printed Ultem 9085 structural properties [1]. A MarkOne 3D printer (Markforged Company, Watertown, MA, USA) was used for 3D printing continuous carbon fiber Nylon composite specimens [2]. The tensile strength and stiffness of the continuous fiber 3D printed parts were compared with the short fiber reinforced Nylon 3D printed parts [2]. The multifunctional aspects of the additively manufactured multifunctional continuous carbon fiber composites are not addressed [2]. Evaluation and prediction of the tensile strength of continuous fiber-reinforced 3D printed structures using the MarkOne 3D printer were conducted in [3]. While this research is a basis for predicting 3D printed parts’ tensile strength, further investigation is needed to mature the 3D printed parts’ mechanical characterization. This paper does not address other needed structural parameters such as compression, bending, and torsion. Effects on the tensile and flexural properties of fused deposition modeling (FDM) 3D printed thermoplastic matrix carbon fiber reinforced plastic specimens were evaluated experimentally in [4]. It should be noted that, while this paper addresses detailed tensile and flexural assessments of 3D printed specimens, it does not address the 3D printed carbon fiber composites’ multifunctional properties. Moreover, the electrical properties [5] of the 3D printed specimens are also not addressed [5]. While the influence of load eccentricity on the behavior of thin-walled compressed composite structures that were manufactured using the traditional methods was conducted, this paper did not address the experimental investigation of the additively manufactured continuous carbon fiber composites for multifunctional electro-tensile properties evaluation [6]. A structural composites battery integration into systems using a traditionally manufactured structural composites battery is presented in [7]. Structural composite battery panels developed in [7] are found to be an integrated power-harvesting platform for a 1U CubeSat frame to supplement or replace interior, external battery packs [7]. AM processes have benefits over the use of traditional manufacturing processes in aerospace applications.

Continuous carbon fiber-reinforced polymer is manufactured using the FDM AM technique [8]. Flexural and tensile strength are evaluated. A comparison of the flexural strength of continuous carbon fiber and chopped carbon fiber-reinforced composites was conducted. A monofunctional structural performance of additively manufactured carbon fiber composites is addressed, and the comparison of the flexural properties of continuous carbon fiber composites and chopped fiber composites are also presented. The multifunctional properties are not addressed in this investigation. FFF technique, also known as the material extrusion AM method, is employed to manufacture continuous carbon and glass fiber-reinforced thermoplastic composites coupons [9]. Its microstructural characteristics with the resulting tensile, flexural, and quasi-static indentation characteristics of the additively manufactured coupons were examined [9]. The extrusion of pre-impregnated continuous carbon and glass fiber composites enables the fabrication of high-performance structural parts due to the high tensile strengths that the continuous fibers provide compared to other pure thermoplastics and short fiber reinforced thermoplastics [9]. Layer-by-layer printing of continuous fiber offers advantages over the traditional manufacturing processes and techniques—like hand lay-up and Automated Fiber-Placement—making the FFF the best option for rapid prototyping optimization [9]. While the FFF of continuous thermoplastic fiber offers great tailorability than the traditional manufacturing methods and processes, this paper’s scope is limited to the FFF’s low deposition rate. This paper does not address the multifunctional properties of the additively manufactured carbon fiber composites. Novel structural supercapacitors fabricated on woven carbon fiber electrodes with glass fiber separators were developed using a vacuum-assisted resin transfer molding process [10]. Test results show a significant increase in energy and power density with a significant increase in tensile strength and modulus [10]. While the device demonstrated some multifunctional performance on the manually fabricated composite structure, this method requires further maturation and research. The additively manufactured multifunctional carbon fiber composite structures’ multifunctional properties are not addressed [10].

Process-dependent factors on the damage and deformations of additively manufactured test coupons are evaluated for uniaxial tensile strength and inverse identification analyses [11]. The effects of varying geometric parameters on the tensile properties of 3D printed composites manufactured by the FFF out of continuous and chopped carbon fiber reinforcement are presented in [12]. The following parameters are varied: infill density and infill patterns of chopped composite material, fiber volume fraction, and printing architecture of continuous fiber reinforcement composites [12]. Characterization of tensile properties and in-situ electrical resistance was conducted on traditionally manufactured specimens made of MXene-coated fibers by employing an Instron 3365 tensile testing machine and connecting them to a Keysight 34461A digital multimeter (Keysight Technologies, Santa Rosa, CA, USA) for electrical resistance measurements in [13]. Simultaneous electrical and mechanical measurements under load are used to study the fiber-matrix interface, the fiber residual compressive stress, and the fiber waviness in carbon fiber composites [14]. The electrical resistance of a traditionally fabricated composite in the through-thickness direction while cyclic tension within the elastic regime is applied in the fiber direction to measure fiber waviness [14]. The nanocomposite specimen’s electrical resistance against the current when the tensile loadings are applied to assess electrical properties of electrospun polyacrylonitrile nanofibers for structural health monitoring purposes [15]. The additively manufactured multifunctional carbon fiber composites coupled with multifunctional properties, in terms of tensile strength and electrical conductivity, are not well addressed to mature for its use on a commercial scale. While the traditional way of conducting monofunctional analyses of aircraft structures comprised of additively manufactured carbon fiber composites, is well understood, the multifunctionality appears to be a new area for exploration. There is a lot of literature on the monofunctional investigation of the monofunctional composites. While there appears to be some good research works on the additive manufacturing of the monofunctional composites and plastics and their monofunctional properties characterization, the experimental investigation of additively manufactured continuous carbon fiber composites for the multifunctional electro-tensile properties is not addressed.

Thus, in this investigation, the FFF 3D printing technique was used to fabricate continuous carbon fiber solid laminate composites test coupons. Multifunctional and coupled aspects of both tensile and electrical parameters of the 3D printed multifunctional continuous carbon fiber solid laminate composites are investigated concurrently. This will help evaluate multifunctional carbon fiber composites’ uses in the aircraft’s primary and secondary structures.

## 2. Materials and Methods

### 2.1. Materials and Additive Manufacturing of Specimen

The test specimens were digitally created as solid models using a CAD tool. The test specimens were manufactured using Markforged’s Continuous Carbon Fiber embedded into Onyx from Markforged Company (Watertown, MA, USA). Since the continuous carbon fiber provides the best strength in the longitudinal direction, it was selected as the raw material. Continuous carbon fiber is also a commonly used composites raw material in aerospace applications due to its high strength-to-weight ratio. The raw materials, design parameters, and process parameters used in this investigation are listed in Table 1. 

The temperature of the Onyx printing nozzle was 275 °C, and that of fiber laying nozzle was 250 °C. A special type of the FFF called as Markforged continuous filament fabrication (CFF) was used. The diameter of the extruded Onyx material was 0.40 mm wall thickness, and the diameter of the extruded fiber material was 0.9 mm wall thickness.

**Figure 1:**
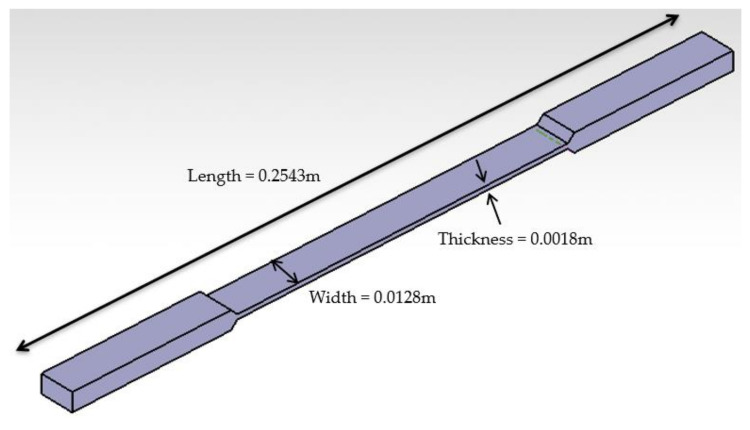
Dimensions of specimen (0.2543 m × 0.0128 m × 0.0018 m).

**Figure 2:**
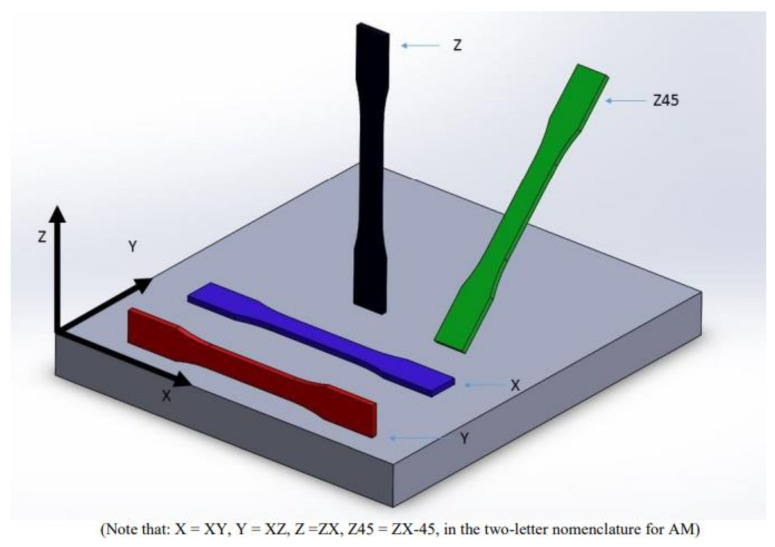
Specimen orientation diagram (permission received from NIAR on 10/29/2021 [19]).

**Figure 3:**
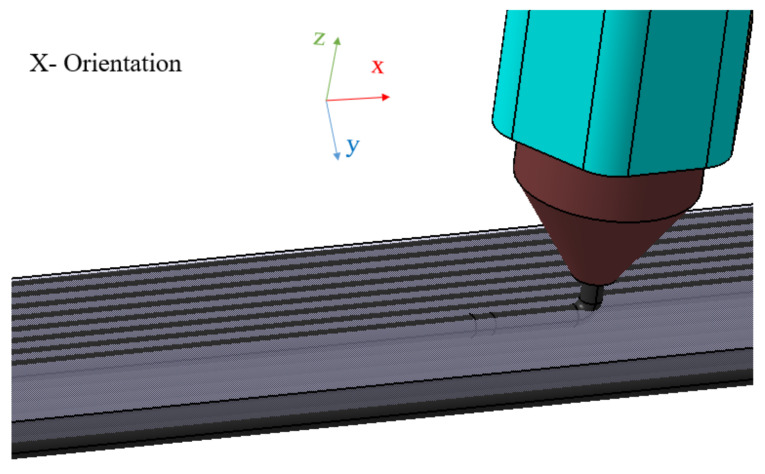
Schematic illustration of the specimen fabrication using an additive manufacturing technique.

**Figure 4:**
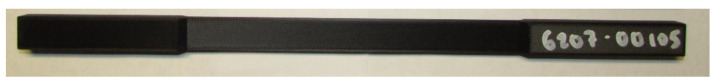
3D printed test specimen from additive manufacturing before instrumentation.

The FFF AM technique was used to fabricate specimens at the RE3DTECH company (Grayslake, IL, USA). Markforged X7 printer was used for the manufacturing of the specimens. A raster angle chosen on the test coupons was 0 degree. Onyx FR and a spool of continuous carbon fiber, both fabricated by Markforged [16], were used as raw materials for this investigation. The Onyx FR was used as a reinforcement with the spool of the continuous carbon fiber composites. The Onyx FR is a flame-resistant Onyx designed for applications where non-flammable parts are needed [17]. The Onyx FR is a UL 94 V-0 Blue Card certified down to a thickness of 3 mm [17]. The particular type of the FFF AM technique, called the Markforged CFF technique [18], was used in this investigation that involved a second nozzle laying down a continuous strand of carbon composite fibers [18]. The average size of the 3D manufactured specimens were 0.2543 × 0.0128 × 0.0018 m^3^ (length × width × thickness). An isometric view of the test coupon is shown in Figure 1. The specimen orientation selected for this investigation was derived from [19]. The specimen orientation diagram and the schematic details of the 3D printed specimen fabrication are shown in Figure 2 and Figure 3, respectively. The 3D printed solid laminate composites test specimen made up of continuous carbon fiber and Onyx FR is shown in Figure 4. The test specimens were 3D printed with tabs on both ends (see Figure 1).

### 2.2. Multifunctional Flexural-Electrical Characterization at Room Temperature Dry

The multifunctional and coupled tensile and electrical properties of additively manufactured continuous carbon fiber composites were simultaneously investigated using an MTS machine (Eden Prairie, MN, USA) [20] per ASTM D3039/D3039M–17 [21] and a Keysight B2987A Electrometer (Keysight Technologies, Santa Rosa, CA, USA ) [22]. The capacity of the load cell on the MTS machine used in this study was 20 kip. The National Institute for Aviation Research (NIAR, Wichita, KS, USA) [23]. 

**Figure 5:**
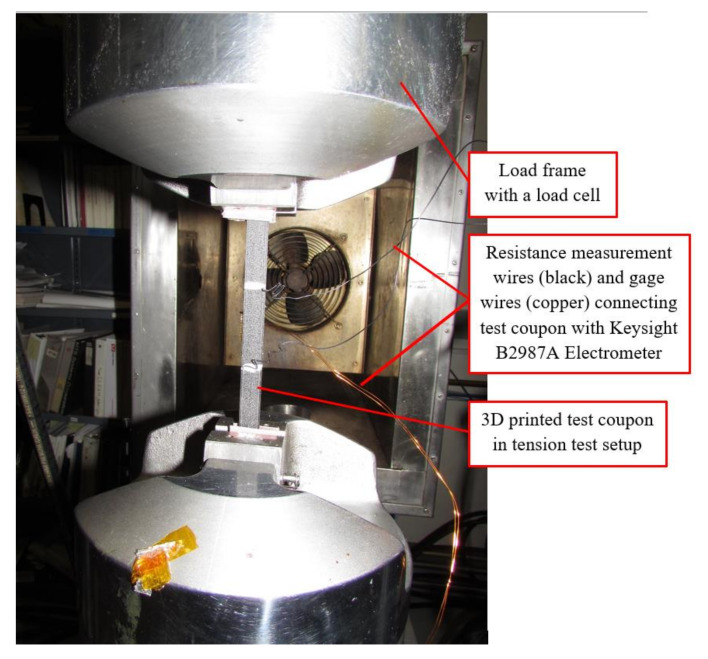
Coupled electro-tensile test set up at RTD.

AGATE-WP3.3-033051-102 was used as a reference [24] and a room temperature dry of 22.5 ± 4.2 °C and ambient relative humidity was selected to evaluate the multifunctional electro-tensile properties of the additively manufactured continuous carbon fiber test coupons. The AGATE stands for the Advanced General Aviation Transport Experiments that was created by the National Aeronautics and Space Administration (NASA) to revitalize the general aviation industry in 1994 [25]. A quasi-static rate of 1.27 mm/min was chosen for this test. The average distance between the two electrical contacts was 50.5 mm. Multifunctional testing, comprising of a concurrent tensile test and electrical tests, were conducted at room temperature dry (RTD) on the test coupons using a multifunctional electro-tensile setup (Figure 5). The purpose of this test was to evaluate the coupled electro-tensile properties of the 3D printed test coupons at the RTD. Resistance of the test coupons was measured after every 445 N until the specimen failure. The selection of 445 N as the interval for resistance measurement was based on the previous dry run test on the test coupons that underwent only structural tensile testing. The results are discussed in detail in the results and discussion section.

## 3. Results and Discussion

**Figure 6:**
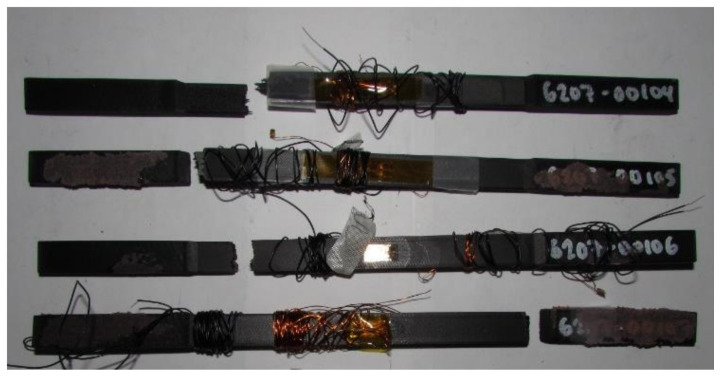
Failure modes of tested coupons from the RTD multifunctional electro-tensile test.

The test coupons were additively manufactured using continuous carbon fiber composites and Onyx FR as raw materials and a Markforged additive manufacturing machine. Electro-tensile properties of additively manufactured continuous carbon fiber solid laminate composites in room temperature dry were investigated using load frame and Keysight B2987A Electrometer (Keysight Technologies). The coupling effects of tensile properties on the electrical properties were assessed. The failure modes of the tested specimens from this multifunctional testing are shown in Figure 6. The failure modes of the test specimens have shown that they are consistent with the failure modes of the traditionally manufactured continuous carbon fiber solid laminate composites. The failure modes and mechanisms play a vital role in the case of advanced material systems like composites due to the anisotropic behavior of the material systems. Thus, the failure modes investigation of composites was conducted experimentally to assess the actual and representative behavior of the composite material systems as they behave in real scenarios. As shown in Figure 6, the failure modes of the test specimens—6207-00105 and 6207-00107 exhibited a lateral (to the longitudinal axis of the specimens) failure mode at the intersection of the grip/tab region and the gage section on the top region of the test specimens. Similarly, the test specimen—6207-00106 exhibited a lateral (to the longitudinal axis of the specimens) failure mode at the intersection of the edge of the gage section and the bottom region of the grip/tab zone towards the top-bottom region of the test specimens. The failure modes of this investigation were compared with the failure modes of the traditionally manufactured carbon fiber composites of [26], and consistent with the failure modes of the 3D printed continuous carbon fiber reinforced thermoplastic composites [27]. It was found that the failure modes of this study were consistent with the failure modes of the traditionally manufactured carbon composites. These findings suggest that the multifunctional tenso-electro properties of the additively manufactured continuous carbon fiber solid laminate composite test specimens are in harmony with the failure modes of the traditionally manufactured carbon composites and that of the 3D printed monofunctional carbon composites. Determination of the failure loads of the test specimens in tensile testing in conjunction with the assessment of the electrical property of the test coupons plays a vital role in the evaluation of the multifunctional tenso-electro properties of the 3D printed test specimens. The failure modes of this study that was found to be consistent with the failure modes of the traditionally manufactured carbon fiber composites, have also suggested that the fibers of the 3D printed multifunctional continuous carbon fiber composites carried the loads along the direction of the fibers.

**Figure 7:**
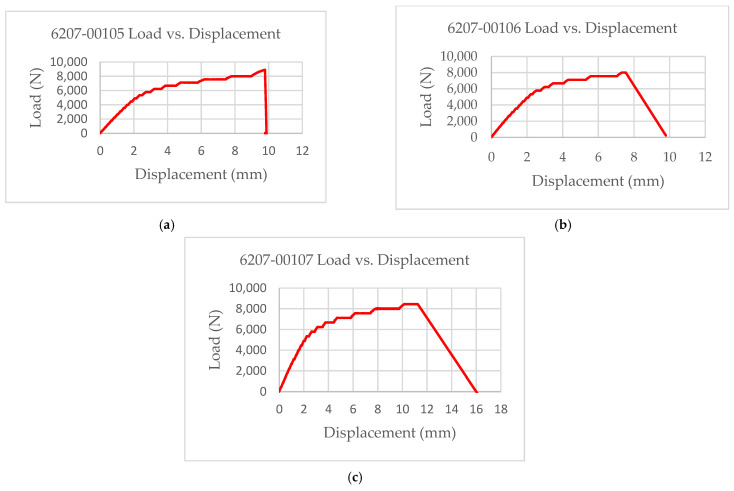
Load versus displacement characteristics of multifunctional test specimens: (**a**) 6207-00105; (**b**) 6207-00105; (**c**) 6207-00107.

**Figure 8:**
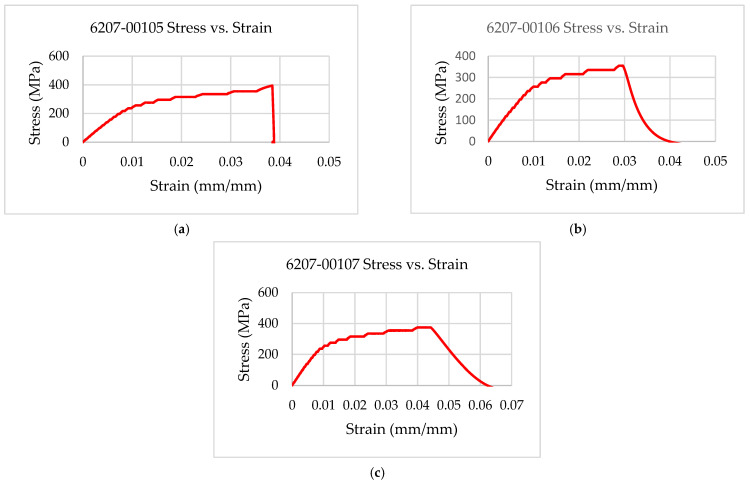
Strain-strain characteristics of multifunctional test specimens: (**a**) 6207-00105; (**b**) 6207-00106; (**c**) 6207-00107.

**Figure 9:**
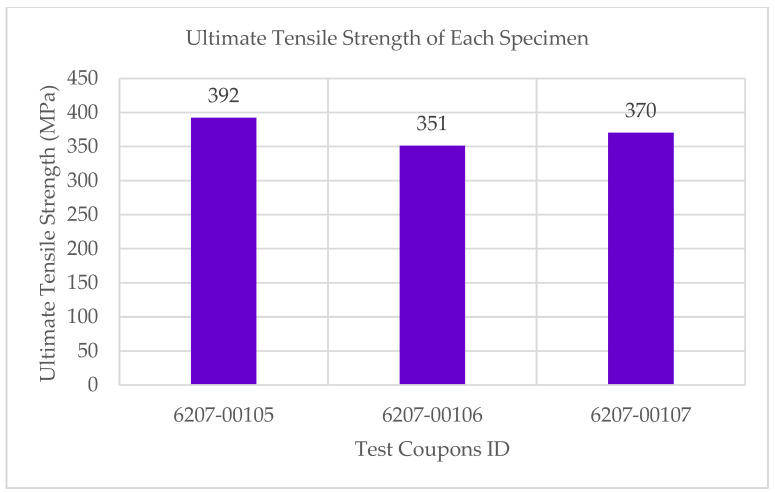
Ultimate tensile strength comparison of test coupons.

**Figure 10:**
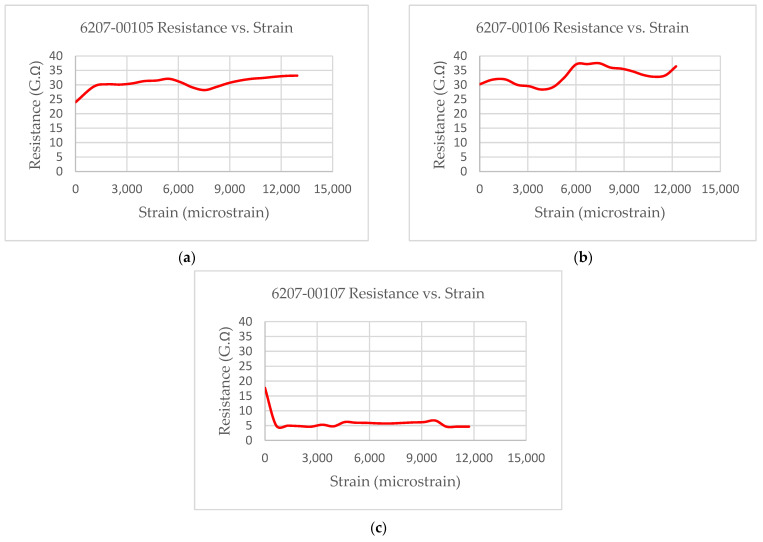
Multifunctional electro-tensile properties of specimens: (**a**) 6207-00105; (**b**) 6207-00106; (**c**) 6207-00107.

**Figure 11:**
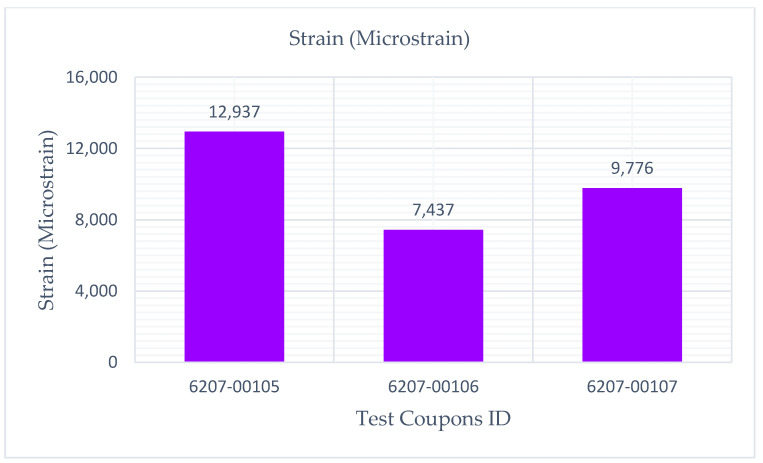
Strain comparison of test coupons corresponding to associated maximum resistance values.

**Figure 12:**
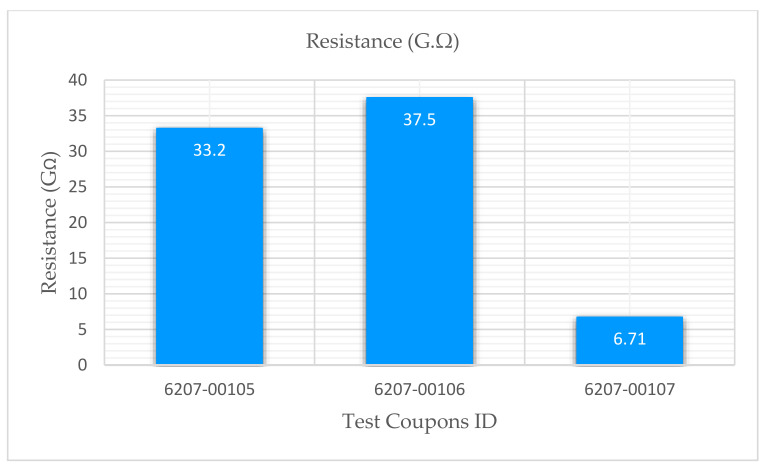
Maximum resistance comparison of test coupons.

**Figure 13:**
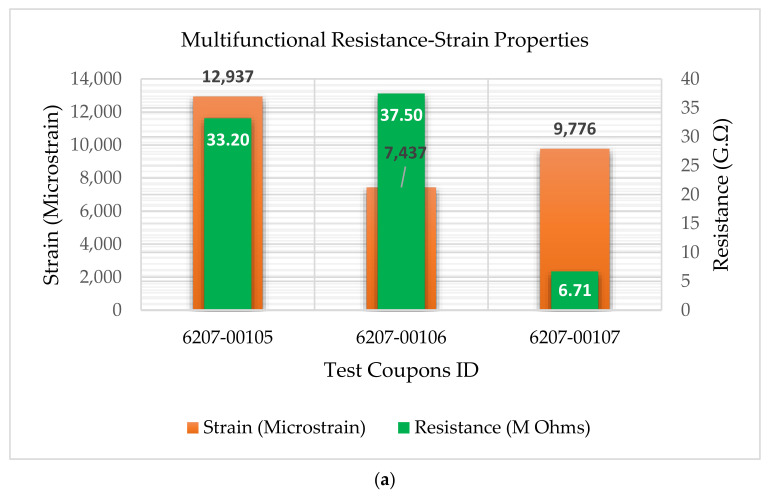

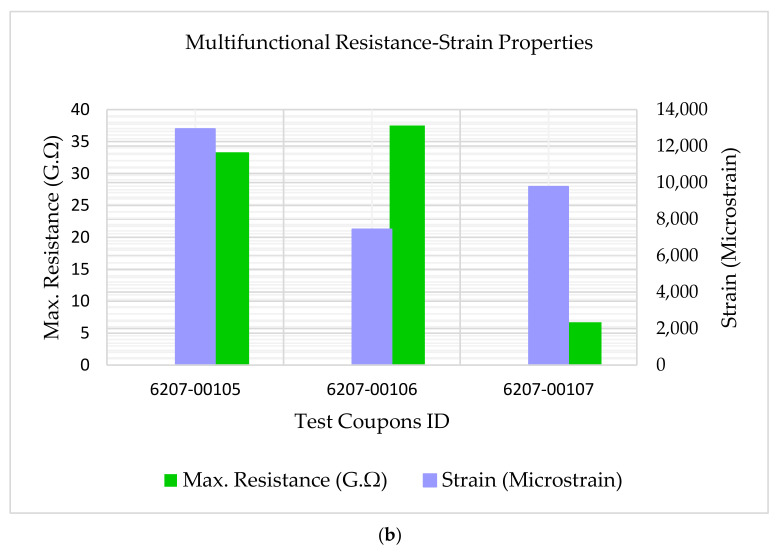
Coupled multifunctional resistance-strain properties comparison of test coupons: (**a**) superimposition of resistance and strain; (**b**) side-by-side comparison.

The experimental results showed that the test coupons exerted a maximum ultimate tensile strength of 392 MPa with an associated maximum load of 8907 N. The electro-tensile response of additively manufactured multifunctional and continuous carbon fiber solid laminate composites is shown in Figure 7, Figure 8, Figure 9 and Figure 10. The load versus displacement behavior of the test coupons is shown in Figure 7. Similarly, stress versus strain is shown in Figure 8, and resistance versus strain is depicted in Figure 10. The nature of the plots obtained in Figure 7 as the load versus displacement characteristics of the multifunctional test specimens are found to be consistent with the load-displacement curve of the traditionally manufactured traditional composites and to the load-displacement plot of the 3D printed monofunctional carbon fiber composites. Similarly, the nature of the stress-strain characteristics of the multifunctional test specimens as shown in Figure 8 are observed to be consistent with the stress-strain relationship plot of the traditionally manufactured traditional composites and to the load-displacement plot of the 3D printed monofunctional carbon fiber composites. The nature of the plots for the load versus displacements of the test coupons as shown in Figure 7 is in agreement with the nature of the plots obtained in the National Center for Advanced Materials Performance (NCAMP) Test Report Number: CAM-RP-2018-013 Rev A [28]. The experimental results of the test specimens are summarized in Table 2. The average ultimate tensile strength of the test coupons was 371 MPa with a corresponding average maximum load of 8459 N. The maximum resistance of the 3D printed continuous carbon fiber solid laminate test coupons was 37.5 G·Ω as the multifunctional property. Table 3 provides the summary of the maximum resistance of test coupons. Based on the values of the maximum resistance obtained for the test coupons from the multifunctional testing and as shown in Table 3, the third value can be considered as an outlier. Since the other two values for the two other test coupons appear to be nicely grouped and close to each other, the maximum resistance value corresponding to the third test coupon appear to be an outlier. The strain corresponding to the peak resistance value was equal to the failure strain in test coupon 6207-00105. Similarly, the strain at peak resistance compared to the respective failure strain was 39.23% lower than the corresponding failure strains for test coupon 6207-00106. The strain associated with the peak resistance value for test coupon 6207-00107 was 19.85% lower than the corresponding failure strain for test coupon 6207-00107. The resistance values during the tests were higher than the residual resistance values for the two test coupons—6207-00105 and 6207-00106. Whereas, for test coupon 6207-00107, the residual resistance value (17.7 G·Ω) was higher than the values recorded during the tests (6.71 G. Ω, 163.79% lower than 17.7 G·Ω). This may be attributed to material and manufacturing defects. Ultimate tensile strength comparison of the test coupons is presented in Figure 9. As shown in Figure 9, the resistance behavior of the test coupons appeared to be reasonably stable and consistent with the linear and non-linear structural deformation of the 3D printed carbon fiber test coupons. A slight fluctuation in the resistance versus strain plots was attributed to the breaking of the external onyx coating from the solid continuous carbon fiber strand in the test coupons. As the tensile effects of the outer layer of the 3D printed coupons resulted in the onset and propagation of the microcracks, the inner layers of the test coupons started to affect the resistance measurements progressively. As shown in Figure 8, the maximum value of the ultimate tensile strength as 392 MPa was obtained for test coupon—6207-00105, and the minimum value of the ultimate tensile strength as 351 MPa was for test coupon—6207-00106. In addition to the nature of the plots obtained from load-displacement (on Figure 7) and stress-strain behaviors (on Figure 8) of the test coupons, the nicely grouped values of the ultimate tensile strength comparison of the multifunctional test coupons obtained from the multifunctional testing procedures suggest that this study’s findings are consistent with the values of the monofunctional composites (traditionally manufactured and 3D printed). The nature of the plots for the stress-strain behaviors of the test coupons as shown in Figure 8 is in harmony with the nature of the plots obtained in the NCAMP Test Report Number: CAM-RP-2018-013 Rev A [28]. This unique behavior of the electrical property and structural phenomena called multifunctional behavior, as shown in Figure 10, highlights a small rise and decrease in resistance along with the strain levels as the test coupons underwent simultaneous multifunctional electro-tensile testing. This small rise and decrease in resistance values along the different strain levels during the test signify that the void contents and delamination defects of the 3D printed continuous carbon fiber test coupons are negligible compared to the traditionally manufactured solid laminates. The lack of delamination and voids on the 3D printed coupons is an enhancing factor for the multifunctional properties of the continuous carbon fiber composites for aerospace applications. The failure mechanisms of the 3D printed specimens shown in Figure 6 infer that the electro-tensile multifunctional performance is similar to the failure modes of the traditional hand-laid up composites. Thus, the electro-tensile properties of additively manufactured continuous fiber composites in room temperature dry investigated through this study are found to be appropriate for aerospace use cases. Figure 11 shows the strain comparison of the test coupons corresponding to the maximum resistance values. Monofunctional representation of the 3D printed multifunctional tenso-electric properties of the multifunctional continuous carbon fiber solid laminate composites is shown in Figure 11 as the strain comparison of the test coupons corresponding to the associated maximum resistance values. The maximum value of the strain comparison of the test coupons is 12,936.67 microstrain, and the minimum of the strain comparison of the test coupons is 7436.51 microstrain. Similarly, Figure 12 shows the maximum resistance comparison of the test coupons. The monofunctional depiction of the multifunctional electro-tensile performance of the 3D printed multifunctional continuous carbon fiber composites is shown in Figure 12 as the maximum resistance values comparison of the test coupons. As shown in Figure 12, the maximum of the maximum resistance values for the test coupons is 37.5 G·Ω, and the least of the maximum resistance values is 6.71. The difference is values of the test results of the test coupons may have been contributed by the material and processing defects. The experimental test results showing the coupling effects of the multifunctional resistance-strain properties comparison of the test coupons are depicted in Figure 13. Figure 13 shows the superimposition of the Figure 11 and Figure 13 as the representation of the multifunctional properties of the 3D printed multifunctional continuous carbon fiber solid laminate composites. Figure 13 also shows the correlation of the strain in microstrain and the maximum resistance values in G·Ω of the test coupons obtained from the multifunctional test procedures of the multifunctional carbon composites.

## 4. Conclusions

The multifunctionality of 3D printed continuous carbon fiber composites is not well investigated due to their relative infancy. While the advanced carbon composites are not new to the research community, the multifunctionality characterization for electro-tensile properties of the multifunctional 3D printed continuous fiber composites is new and was explored in this research. This research designed, developed, tested, and analyzed the electrical conductivity effect on the 3D printed multifunctional continuous carbon fiber composites’ tensile properties. The proposed method addressed the integrated characterization of the multifunctional carbon fiber composite structures’ electrical and tension properties. The experimental results showed that the coupons exerted a maximum ultimate tensile strength of 392 MPa and associated maximum load of 8907 N. The average ultimate tensile strength of 371 MPa, average maximum load of 8459 N, and the maximum electrical resistance of 37.5 G·Ω, was observed as the multifunctional property. The electrical resistance of the coupons was considerably stable and consistent with the structural deformation of the 3D printed continuous carbon fiber test coupons. Slight fluctuation in the resistance versus strain plots may be attributed to the breaking of the external onyx coating from the solid continuous carbon fiber strand in the test coupons. The contacts amongst the onyx coatings and the inner continuous carbon fiber strands touched amongst themselves during the tensile testing of the coupons. This phenomenon resulted in a slight fluctuation in the electrical resistance versus strain and also initiated the onset and propagation of the microcracks. This unique behavior, called multifunctional behavior, highlights a small rise and decrease in resistance along with the strain levels due to simultaneous multifunctional electro-tensile testing. A small fluctuation in electrical resistance values along the strain levels signified the void contents and delamination defects of the coupons are negligible compared to the traditionally manufactured solid laminate composites. Thus, the lack of delamination and voids on the 3D printed coupons compared with the traditionally manufactured composites is an enhancing factor for the multifunctional properties of the continuous carbon fiber composites for aerospace applications. The consistent failure modes and their mechanisms of the specimens implied the electro-tensile multifunctional performance is similar to the traditional composites. There are still not enough publications addressing information and knowledge on multifunctional continuous carbon fiber composites production by the FFF and on their mechanical, physical and electrical characterizations. There are a lot of literature on the monofunctional investigation of the monofunctional composites. While there appears to be some good research works on the additive manufacturing of the monofunctional composites and plastics and their monofunctional properties characterization, the experimental investigation of additively manufactured continuous carbon fiber composites for the multifunctional electro-tensile properties is not addressed. Thus, this highlights the novelty of this study.

The experimental electro-tensile properties of the additively manufactured continuous fiber composites at room temperature dry investigated in this study show promising application towards aerospace utilization due to their inherent multifunctional properties. In addition, the observed failure modes and mechanisms were found to be consistent when compared to their traditional monofunctional composites counterparts. Future direction of this research work can include validation of this study with analysis. The generated tenso-electric multifunctional properties from the multifunctional testing of the multifunctional continuous carbon fiber composites from this study can be employed on analysis in future research investigation.

## Figures and Tables

**Table 1:** AM, materials, and processing.

Items	Information
Make of AM machine	Markforged company (Watertown, MA, USA)
Model of AM machine	Markforged X7 (Markforged company, Watertown, MA, USA)
Series of AM machine	Industrial Composite 3D Printers
AM process/type	Special type of the FFF called as Markforged CFF
Material specifications	Markforged manufactured Onyx FR with continuous carbon fiber (solid Onyx as outside walls and inside core was continuous carbon fiber)
Finish	Markforged CFF with 50% fiber reinforcement
No. of extruders	2
Raster angle of test coupons	0 degree
Temperature of Onyx Printing Nozzle	275 °C
Temperature of Fiber-Laying Nozzle	250 °C
Diameter of the extruded Onyx material	0.40 mm Wall Thickness
Diameter of the extruded fiber material	0.9 mm Wall Thickness
Surface Finish on 3D printed specimens	0.125 mm Layer Height
Raw materials	Onyx (Markforged Material) + Carbon Fiber

**Table 2:** Summary of the experimental results of the test coupons.

Coupon ID	Maximum Load, P^max^ (N)	Ultimate Tensile Strength, F^tu^ (MPa)
6207-00105	8907	392
6207-00106	8017	351
6207-00107	8454	370
Average	8459	371
Standard Deviation	445.02	20.52
Coefficient of Variation (COV)	5%	6%

**Table 3:** Summary of the maximum resistance of test coupons.

Coupon ID	Maximum Resistance (G. Ω)
6207-00105	33.2
6207-00106	37.5
6207-00107	6.7
Average	25.80
Standard Deviation	16.67

## Data Availability

Not applicable.

## References

[1] Zhang Y., Moon S. (2021). The Effect of Annealing on Additive Manufactured ULTEM^™^ 9085 Mechanical Properties. Materials.

[2] Blok L.G., Longana M.L., Yu H., Woods B.K.S. (2018). An investigation into 3D printing of fibre reinforced thermoplastic composites. Addit. Manuf..

[3] Melenka G.W., Cheung B.K.O., Schofield J.S., Dawson M.R., Carey J.P. (2016). Evaluation and prediction of the tensile properties of continuous fiber-reinforced 3D printed structures. Compos. Struct..

[4] Ning F., Cong W., Qiu J., Wei J., Wang S. (2015). Additive manufacturing of carbon fiber reinforced thermoplastic composites using fused deposition modeling. Comp. Part B Eng..

[5] D’Aloia A.G., Proietti A., Bidsorkhi H.C., Tamburrano A., De Bellis G., Marra F., Bregnocchi A., Sarto M.S. (2018). Electrical, Mechanical and electromechanical properties of graphene-thermoset polymer composites produced using acetone-dmf solvents. Polymers.

[6] Wysmulski P., Debski H., Falkowicz K., Rozylo P. (2019). The influence of load eccentricity on the behavior of thin-walled compressed composite structures. Compos. Struct..

[7] Moyer K., Meng C., Marshall B., Assal O., Eaves J., Perez D., Karkkainen R., Roberson L., Pint C.L. (2020). Carbon fiber reinforced structural lithium-ion battery composite: Multifunctional power integration for CubeSats. Energy Storage Mater..

[8] Yu T., Zhang Z., Song S., Bai Y., Wu D. (2019). Tensile and flexural behaviors of additively manufactured continuous carbon fiber-reinforced polymer composites. Compos. Struct..

[9] Goh G.D., Dikshit V., Nagalingam A.P., Goh G.L., Agarwala S., Sing S.L., Wei J., Yeong W.Y. (2018). Characterization of mechanical properties and fracture mode of additively manufactured carbon fiber and glass fiber reinforced thermoplastics. Mater. Des..

[10] Deka B., Hazarika A., Kwon O., Kim D., Park Y.-B., Park H.W. (2017). Multifunctional enhancement of woven carbon fiber/ZnO nanotube-based structural supercapacitor and polyester resin-domain solid-polymer electrolytes. Chem. Eng. J..

[11] Kerekes T.W., Lim H., Joe W.Y., Yun G.J. (2019). Characterization of process–deformation/damage property relationship of fused deposition modeling (FDM) 3D-printed specimens. Addit. Manuf..

[12] Naranjo-Lozada J., Ahuett-Garza H., Castañón P.O., Verbeeten W.M., Sáiz-González D. (2019). Tensile properties and failure behavior of chopped and continuous carbon fiber composites produced by additive manufacturing. Addit. Manuf..

[13] Hatter C.B., Sarycheva A., Levitt A., Anasori B., Nataraj L., Gogotsi Y. (2020). Electrically Conductive MXene-Coated Glass Fibers for Damage Monitoring in Fiber-Reinforced Composites. C.

[14] Wang X., Fu X., Chung D.D.L. (1998). Electromechanical study of carbon fiber composites. J. Mater. Res..

[15] Alarifi I., Alharbi A., Khan W.S., Swindle A.L., Asmatulu R. (2015). Thermal, Electrical and Surface Hydrophobic Properties of Electrospun Polyacrylonitrile Nanofibers for Structural Health Monitoring. Materials.

[16] Markforged Markforged Materials. https://markforged.com/about/company.

[17] Markforged Markforged: Composites Material Datasheet. http://static.markforged.com/downloads/composites-data-sheet.pdf.

[18] Markforged Markforged: All Your Questions about 3D Printing Answered. https://markforged.com/resources/blog/3d-printing.

[19] Lovingfoss R., Clarkson E. (2019). Stratasys Certified ULTEM 9085 Fortus 900mc Additively Manufactured Polymer Material Qualification Statistical Analysis Report.

[20] MTS Company MTS Company: Materials Test Systems. https://www.mts.com/en/products/materials.

[21] ASTM (2017). ASTM D3039/D3039M—17, Standard Test Method for Tensile Properties of Polymer Matrix Composite Materials.

[22] Keysight Technologies Keysight Technologies: Keysight B2987A Electrometer. https://www.keysight.com/us/en/support/B2987A/electrometer-high-resistance-meter-0-0-1fa-battery.html.

[23] NIAR National Institute for Aviation Research (NIAR). https://www.wichita.edu/research/NIAR/.

[24] NIAR (2001). AGATE: B—Basis Design Allowables for Epoxy—Based Prepreg.

[25] NASA Advanced General Aviation Transport Experiments (AGATE). https://www.nasa.gov/centers/langley/news/factsheets/AGATE.html.

[26] De Paiva J.M.F., Mayer S., Rezende M.C. (2006). Comparison of tensile strength of different carbon fabric reinforced epoxy composites. Mater. Res..

[27] Van Der Klift F., Koga Y., Todoroki A., Ueda M., Hirano Y., Matsuzaki R. (2016). 3D Printing of Continuous Carbon Fibre Reinforced Thermo-Plastic (CFRTP) Tensile Test Specimens. Open J. Compos. Mater..

[28] National Institute for Aviation Research (NIAR) (2020). Stratasys Certified ULTEMTM 9085 Fortus 900mc Additively Manufactured Polymer Material Qualification Data Report.

